# Incomplete but Infectious Vaccinia Virions Are Produced in the Absence of Oncolysis in Feline SCCF1 Cells

**DOI:** 10.1371/journal.pone.0120496

**Published:** 2015-03-23

**Authors:** Suvi Parviainen, Karoliina Autio, Markus Vähä-Koskela, Kilian Guse, Sari Pesonen, Thomas J. Rosol, Fang Zhao, Akseli Hemminki

**Affiliations:** 1 Cancer Gene Therapy Group, Department of Pathology and Transplantation Laboratory, Haartman Institute, University of Helsinki, Helsinki, Finland; 2 Department of Equine and Small Animal Medicine, Faculty of Veterinary Medicine, University of Helsinki, Helsinki, Finland; 3 Department of Veterinary Biosciences, College of Veterinary Medicine, The Ohio State University, Columbus, Ohio, The United States of America; 4 Advanced Microscopy Unit, Department of Pathology, Haartman Institute, University of Helsinki, Helsinki, Finland; University Hospital of Navarra, SPAIN

## Abstract

Vaccinia virus is a large, enveloped virus of the poxvirus family. It has broad tropism and typically virus replication culminates in accumulation and lytic release of intracellular mature virus (IMV), the most abundant form of infectious virus, as well as release by budding of extracellular enveloped virus (EEV). Vaccinia viruses have been modified to replicate selectively in cancer cells and clinically tested as oncolytic agents. During preclinical screening of relevant cancer targets for a recombinant Western Reserve strain deleted for both copies of the thymidine kinase and vaccinia growth factor genes, we noticed that confluent monolayers of SCCF1 cat squamous carcinoma cells were not destroyed even after prolonged infection. Interestingly, although SCCF1 cells were not killed, they continuously secreted virus into the cell culture supernatant. To investigate this finding further, we performed detailed studies by electron microscopy. Both intracellular and secreted virions showed morphological abnormalities on ultrastructural inspection, suggesting compromised maturation and morphogenesis of vaccinia virus in SCCF1 cells. Our data suggest that SCCF1 cells produce a morphologically abnormal virus which is nevertheless infective, providing new information on the virus-host cell interactions and intracellular biology of vaccinia virus.

## Introduction

Vaccinia virus (VV) is a large, enveloped virus belonging to the poxvirus family. Currently, the virus can only be found as a laboratory strain used for the study of poxviruses or cancer treatment. It has a linear, double-stranded DNA genome approximately 190 kbp in length encoding for approximately 250 genes [1]. The most studied member of the poxvirus family is variola, the causative agent of smallpox and vaccinia virus is well-known for its role as a vaccine used in the Smallpox Eradication Program led by World Health Organization [2, 3]. Since the eradication of smallpox, VV has been studied as a viral vector for the development of oncolytic virus therapies, immunotherapies, and as the development of next-generation smallpox vaccines [4].

Replication-competent oncolytic vaccinia viruses have shown great promise as a potential cancer treatment [5]. Over the past decade, hundreds of cancer patients have been treated with vaccinia virus in clinical trials, evaluating several different genetically engineered vaccinia viruses [6]. The development of virotherapeutics has led to the use of safety- and selectivity-enhanced viruses [7]. Vvdd, a recombinant VV with deletions of the viral thymidine kinase (tk) and vaccinia growth factor (vgf) genes, has shown significantly improved safety profile relative to the wild-type (wt) or single-deleted mutants while still being able to maintain its oncolytic potency [8].

Importantly, because of its broad tropism, vaccinia virus is also being developed for virotherapy in domestic animals and is currently being tested in clinical trials at least in pet dogs [9]. Conversely, large animal trials may serve as gateways into human cancer patients, and thus, we and others have been interested in developing novel oncolytics for both humans and pets. During screening of several human and animal cancer cell lines for permissiveness, we discovered unusual infection properties in a particular feline squamous carcinoma (SCC) cell line, SCCF1. Recombinant oncolytic vaccinia virus was able to enter the cells and direct early gene expression. Instead of oncolysis, in confluent SCCF1 monolayers vaccinia virus persisted for up to two weeks without completely lysing the cells. Moreover, persistently infected SCCF1 cells continuously secreted infectious vaccinia particles, confirmed by plaque assay on human A549 cells. Thin section analysis revealed only immature virus particle formation in the cytoplasm of the infected SCCF1 cells and further analysis of infected cell culture supernatants showed particles with discontinuous membranes, arguing that SCCF1 cells do not support the final steps of vaccinia virus particle formation.

## Material and Methods

### Cell lines

Feline squamous cell carcinoma cell line (SCCF1) was kindly provided by Dr. T. Rosol (Ohio State University, US). SCCF1 has been developed from a laryngeal SCC of a cat. Keratinocytes were maintained in culture for greater than 50 passages. SCCF1 had strong cytokeratin immunohistochemical staining, weak vimentin staining and no p53 staining [10]. Also african green monkey kidney epithelial cells (Vero) and adenocarcinomic human alveolar basal epithelial cell line (A549) were used in the study. Vero and A549 cell line were purchased from American Type Cell Culture Collection (ATCC) (Manassas, VA USA) and all cell lines were maintained in conditions recommended by ATCC (A549 cells) or by the provider of the cell line (SCCF1). A549 cells were maintained in Dulbecco´s Modified Eagle Medium (DMEM) with 1 g/l glucose Lonza (Veviers, Belgium) supplemented with 10% fecal calf serum (FCS), 1% L-glutamine and 1% penicillin-streptomycin (P-S) and SCCF1 cells were maintained in DMEM with 4.5 g/l glucose Lonza (Veviers, Belgium) supplemented with 10% FCS, 1% L-glutamine and 1% P-S.

### Viruses

The viruses used in the study were all cancer specific double deleted vaccinia viruses (vvdd) of Western Reserve strain and they have total deletion of vaccinia growth factor (vgf) and a partial deletion of thymidine kinase (tk) gene. All viruses have also insertion of *lacZ* gene in vgf site coding inactive but immunogenic beta-galactosidase enzyme. Wild-type control was not included in the studies due to the biosafety regulations. In addition, vvdd-luc has insertion of luciferase gene and vvdd-tdTomato encodes fluorescent protein tdTomato. The viruses were engineered as previously described [11, 12]. Vvdd-luc was amplified on Vero cells and vvdd-tdTomato in A549 cells. Sucrose cushion was used in purification and titers were determined by standard plaque assay as described previously [11].

### In vitro cytotoxicity assay

Cell viability after viral infection was measured using a colorimetric cell lysis test (MTS). Ten thousand cells per well were plated on 96-well plates in growth medium (GM) supplemented with 5% fetal calf serum (FCS). Confluent cell layers were infected in triplicates with different concentrations of virus for 1 hour in 2% FCS GM. After that, 5% FCS GM was added into wells and cells were incubated until cell viability was measured by MTS according to manufacturer’s instructions (Cell Titer 96 AQ_ueous_ One Solution Proliferation Assay, Promega, Madison, WI). Inactivated vaccinia virus was used as a non-replicating control, inactivation of the virus was done as described previously [11].

### In vitro transduction assay

Transduction of vvdd-luc was performed plating 100,000 cells per well in a 24-well plate in 5% FCS GM. After 48 h incubation, the cells were washed once with 2% FCS GM and infected in triplicates with different concentrations of virus in 2% FCS GM for 30 min. Infected cells were washed once with 10% FCS GM and incubated for 4 h. Finally, cells were lysed and luciferase activity was measured according to the manufacturer’s instructions (Luciferase Assay System, Promega, Madison, WI, USA). Transduction of tdTomato was also confirmed by infecting cells with 0.2, 1 or 5 pfu/cell and visualizing the tdTomato expression 24 hours later with fluorescent microscope.

Presence of viable virus inside the cells was also confirmed by plating 200,000 cells per 24-well in 5% FCS GM. Next day, the cells were infected in triplicates at 0.01 pfu per cell in 2% FCS GM for 1 hour. After infection, 5% FCS GM was added and cells were collected after 24, 48 and 72 h. After 3 freezing-thawing cycles a standard plaque forming assay was performed.

### In vitro clonal assay

Ten thousand and 50,000 cells were plated on 6-well plates and infected 48 h after with 10 pfu per cell for 1 h in 2% FCS GM. Cells were scraped from the wells and collected with supernatant at 24 and 72 h. Different amounts of cells were plated in triplicates on 6 well plates. One or 3 days after plating, colonies were stained with crystal violet and counted.

### Amount and infectivity of the produced virions in vitro

Infected A549 cells and SCCF1 cells were collected and virus was extracted and purified to determine the amount of virus produced by the cells. Also supernatant of the infected SCCF1 cells was collected on different time points and plaque assay was performed to determine the production rate of secreted virions. To study the efficacy of secondary infection, collected supernatant was also used to infect either SCCF1 or A549 cells and transduction assay was performed as described previously.

### Neutralization assay

Supernatant of infected SCCFI cells was collected and incubated with or without NR-417 monoclonal anti-vaccinia antibody (BeiResources, Manassas, VA) for 1 hour in +37°C. Standard plaque assay was performed to determine the amount of neutralized virus.

### Electron microscopy

Two EM specimen preparation methods, negative staining of whole mount viral particle and thin sectioning of resin embedded cells with viruses, were used in this study.

1. Negative staining: this method is used to study surface structures of purified, whole-mount vaccinia viral particles in the supernatant of infected SCCF1 and A549 cells. The method detects viral particle as a whole in 3 dimensions. For asymmetrical particle like vaccinia virus, the orientation is not random, with the largest, flat surface attached and facing up predominantly. To get purified viral particle, supernatant of infected cells was collected and the virus was purified through sucrose cushion as described previously [11]. Viral particles were absorbed on formva-carbon coated EM specimen supporting grids by applying one droplet (10–20 μl) of virus suspension on top of the grids, absorbing for 1 minute. The suspension was then blotted away by Whatman filter paper, one droplet (30–40 μl) of negative stain, 2% Potassium phosphotungstate (KPT), pH 7.2, was added on top of the grids, and stained for 30 seconds. The stain was blotted away and the grids were air-dried for 2 minutes and loaded into electron microscope for observation.
2. Resin embedded thin section electron microscopy: this method is used to study internal structure of viral particle and infected host cells. It can reveal structures of vaccinia viral particles at any orientation without bias as the embedding and cutting direction is random relative to the particles. For doing this, cultured SCCF1 and A549 cells were infected with vaccinia and 48 hours after the infection the cells were fixed on-site in culture dish immediately out of incubator with 2.5% glutaraldehyde in 0.1 M phosphate buffer (pH 7.4) for 30 minutes and the cells were scraped off the dish, collected by centrifuge as a small pellet of 2–3 mm^3^. Fresh glutaraldehyde fixative was added on top of the pellet and fixed further for half an hour. The pellets were then post-fixed by 2% osmium tetroxide for 1 hour, dehydrated in series of ethanol and embedded in LX-112 resin. Ultra-thin sections were cut at thickness of 60–80 nm, mounted on 200 mesh EM specimen supporting grids, stained with uranyl acetate and lead citrate in Leica EMstain automatic stainer (Leica microsystems, Austria) according to the manufacturer´s recommendations and then observed by EM.

Both negative stained specimen and thin sections were observed by using JEM 1400 transmission electron microscope (JEOL, Tokyo, Japan), at 80 KV acceleration voltage. Digital electron micrographs were taken by a side-mounted Morada TEM digital camera (Olympus Soft Imaging Solution GMBH, Munster Germany). Images were processed (brightness and contrast operation only) by iTem software from the camera manufacturer. Adobe Photoshop software was used for annotation.

### Statistical analysis

All values are indicated as mean standard error of the mean (SEM). Differences between groups were calculated using two-tailed Student’s t-test (GraphPrism software). *P*<0.05 was considered statistically significant.

## Results

### In vitro cytotoxicity and transduction of the virus in SCCF1 and A549 cell line

**Fig 1:**
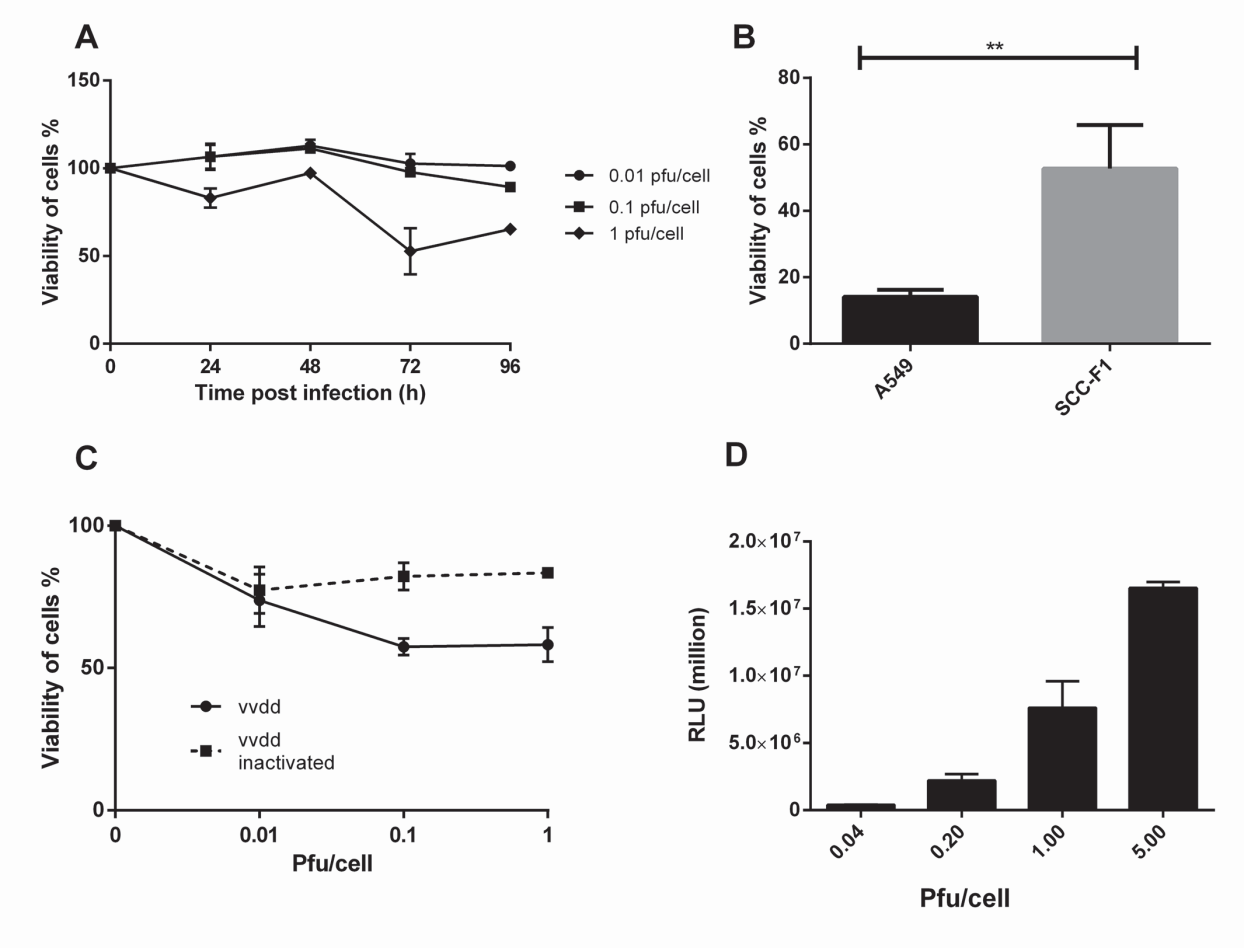
In vitro cytotoxicity and and transduction of vvdd in SCCF1 cells. **A)** SCCF1 cells were infected with vvdd with 0.01, 0.1 or 1 pfu/cell. Viability of cells was measured on day 3. **B)** Compared to A549 cells, SCCF1 cells maintained their viability significantly better on day 3 **C)** When SCCF1 cells formed a tight monolayer before infection, the cells were still alive 10 days after infection. **D)** SCCF1 cell were infected with 0.04, 0.2, 1 or 5 pfu per cell and luciferase expression was measured 4 h postinfection in relative light units.

Vvdd was unable to effectively lyse SCCF1 cells when pfu/cell values 0.01, 0.1 or 1 were used (**Fig. 1A**). With extremely high amount of virus, cytotoxicity could eventually be achieved on day 10 (data not shown). However, vvdd proved significantly more effective (*p* = 0.0073) cytotoxicity in A549 cells compared to SCCF1 cells when both cell lines were infected with 1 pfu/cell and viability was measured on day 3 (**Fig. 1B**). When SCCF1 was growing as an intact monolayer, infected cells were not completely lysed even 10 days after infection (**Fig. 1C**). Successful virus entry and early gene expression in the SCCF1 cells was confirmed by virus-mediated expression of firefly luciferase (**Fig. 1D**). Viable virus was also shown to be located inside the SCCF1 cells by plaque assay (**S1 Fig.**).

### Clonality of the infected cells

Clonal assay was performed in SCCF1 cell lines since they were more resistant for oncolysis caused by vaccinia viruses. One day after infection cell colonies were formed in all samples expect when 10 infected cells were plated. When cells were plated 3 d after infection approximately 50% fewer colonies were identified when 1,000 cells were plated compared to negative control. Results are summarized in S1 Table.

### Quantification and infectivity of the produced virions in vitro

**Fig 2:**
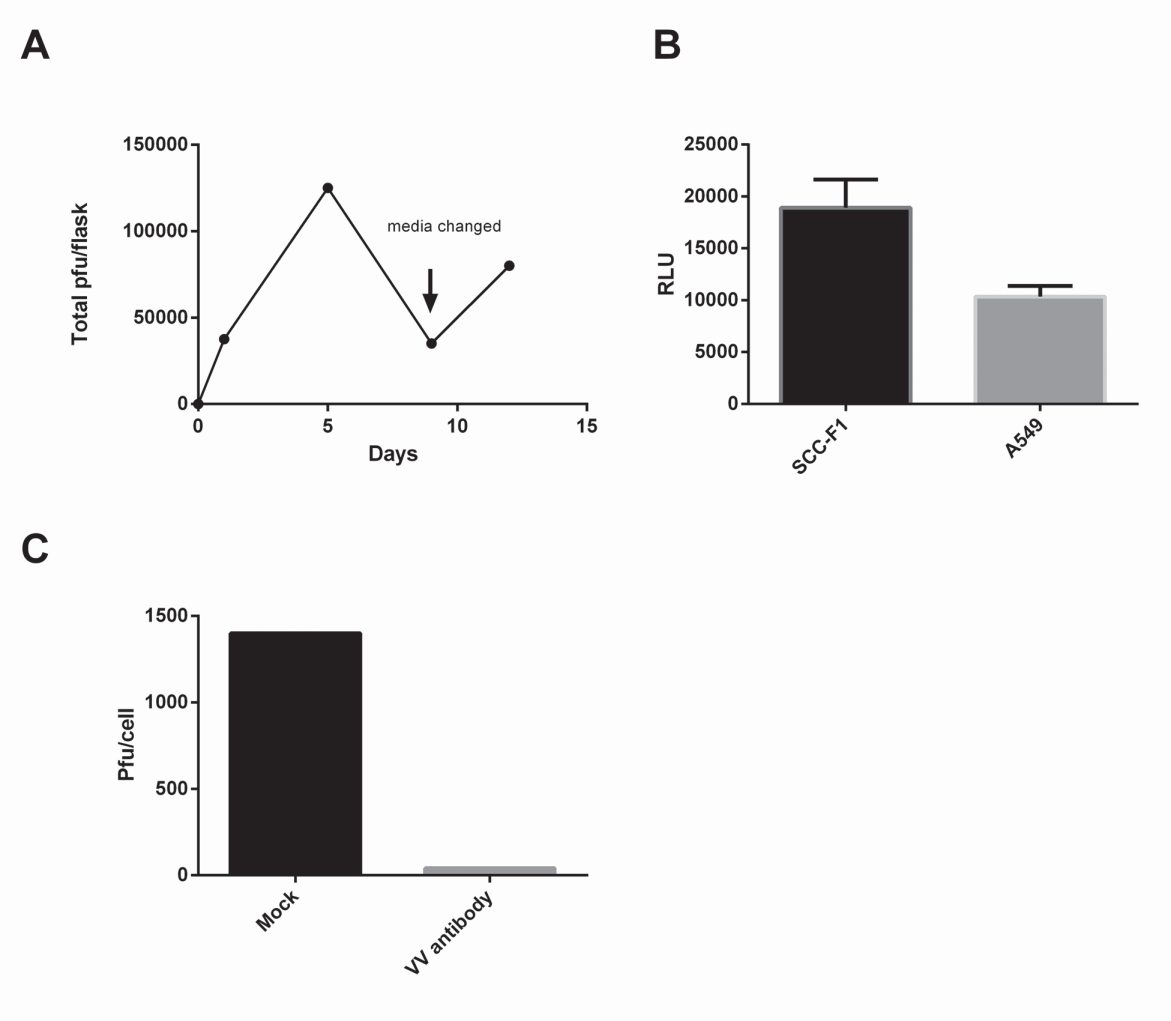
Production of virus in SCC-F1 cells. **A)** Infected SCC-F1 cells constantly produce infectious particles to the supernatant. **B)** Virus released to the supernatant by infected SCCF1 cells was able to re-infect both A549 and SCC-F1 cells. **C)** Viral particles released to the supernatant were completely neutralized with anti-VV antibody.

In order to assess whether vaccinia-mediated reporter gene expression was associated with productive virus replication, we titered chronically infected confluent SCCF1 cell culture supernatant on different time points. We observed low but constant secretion of virus at least up to 12 days post infection (**Fig. 2A**). Secreted particles were also used for re-infecting SCCF1 and A549 cells and in both cell lines these particles were able to cause secondary infection indicating that these produced virions were infectious (**Fig. 2B**). To further characterize the nature of the particles neutralization assay was performed showing that basically all the particles can be recognized and neutralized by anti-vaccinia antibody (**Fig. 2C**).

### Abnormal morphology of vaccinia virions secreted by cat SCCF1 cells

**Fig 3:**
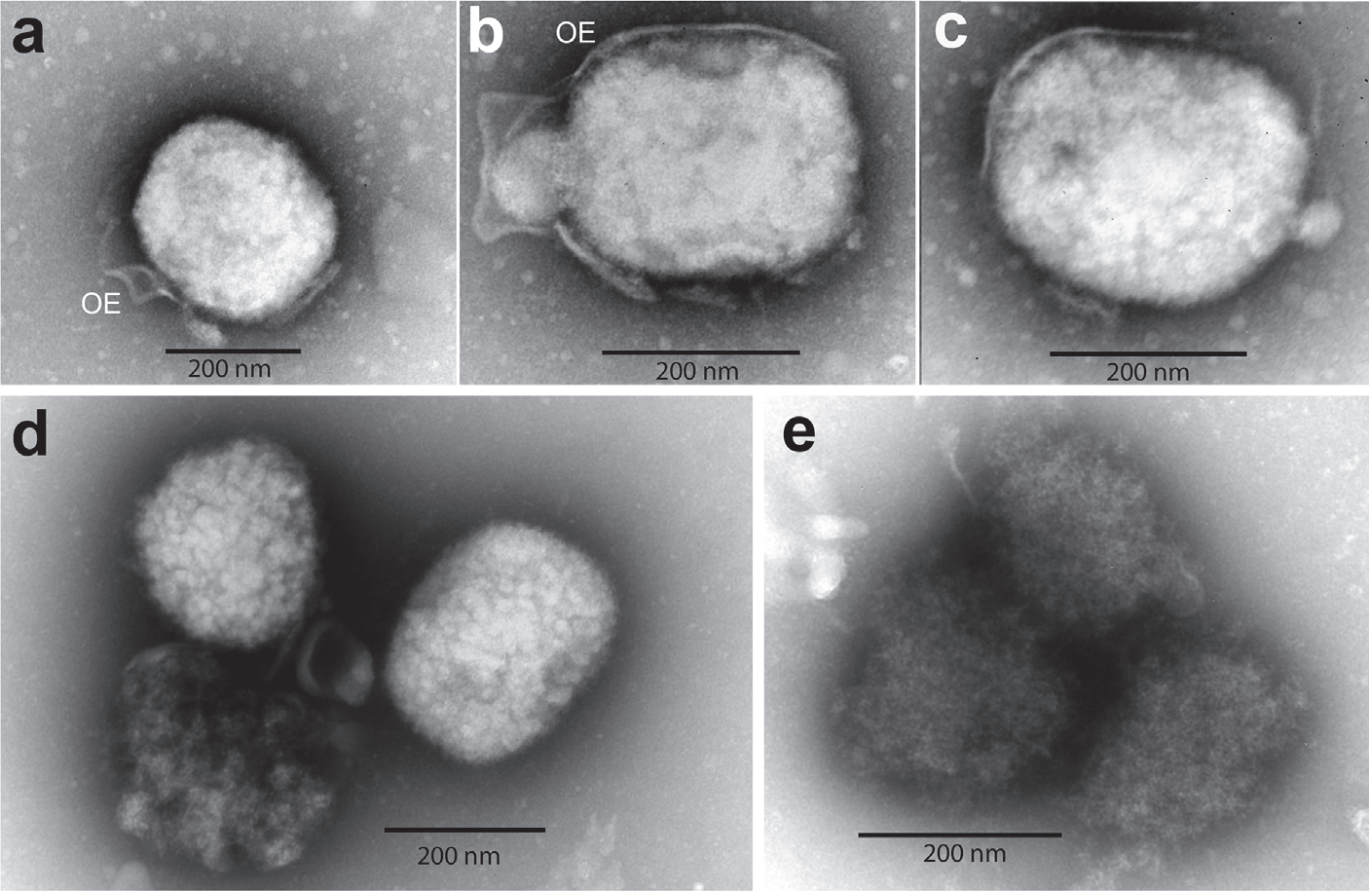
Electron microscopy of negative stained, purified vaccinia viral particles released from cultured cells. Supernatant from infected SCCF1 or A549 cells was collected, purified and negative staining electron microscopy was performed. **A-C:** viral particles produced by A549 cells: mature particles of brick-shaped, with outer envelope (OE) derived from cell membrane. **D-E**: immature vaccinia viral particles produced by SCCF1 cells: the particles are irregular in shape and in electron density. Their internal contents are not tightly packed, no clear boundary or membranous outer envelope is visible.

Electron microscopy on purified, secreted virus particles revealed clear differences between particles produced by SCCF1 versus A549 cells. A549 cells were able to produced mature, enveloped virions with features typical of mature vaccinia viruses (**Fig. 3A, B, C**). All of the viral particles were brick-shaped and are surrounded by an outer envelope (OE). In contrast, all virions produced by SCCF1 cell line were misshaped immature virions. Further, no outer envelopes are detected and particles were loosely packed with irregular surface and irregular staining intensity (**Fig. 3D, E**). Normal ultrastructural organelle morphology of SCCF1 cells is shown in S2 Fig.

### Ultrastructure of viral particles within infected cells revealed by thin section

The replication, maturation and morphogenesis of vaccinia virus within a host cells is a process consisting of successive intermediate steps thus producing a series of middle stage products. Depending on specimen preparation methods employed in electron microscopy, particles with different shapes and contents can be detected (primarily because the preferred orientation and exposed plane of viral particle relative to electron beam in the microscope). Nomenclature of the different particle structures is inconsistent in the literature for historic reason. Moss proposed a simplified naming system for normal, mature poxvirus [13] using only three names for mature, infectious form of the virus including MV (maturate virus), WV (wrapped virus) and EV (extracellular virus). MV is mainly located inside cells excepting released via cell lysis. MV replaces previous name IMV (intracellular mature virus). WV is located either intracellularly or extracellularly and it replaces the old name IEV (intracellular enveloped virus), EEV (extracellular enveloped virus) and CEV (cell associated extracellular enveloped virus). In our study, because more emphasis is put on different immature particles, we still use the old, descriptive nomenclature for better identifying different mid-stage particles.

**Fig 4:**
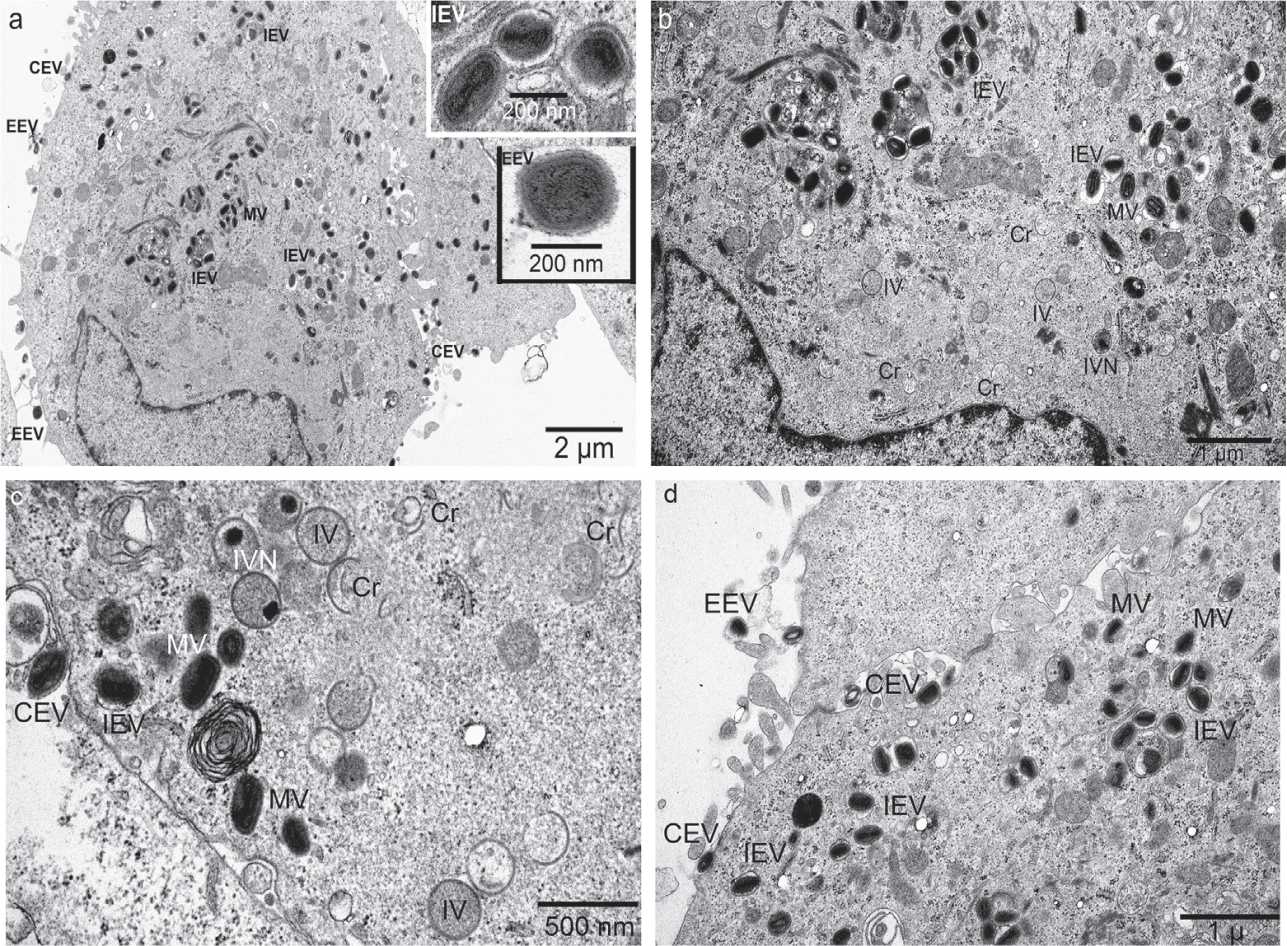
Vaccinia virus replication and maturation in A549 cells. **A-B**: After enter A549 cells, at 48 hours post infection, vaccinia viral particles begin their normal life cycle quickly: form replication centre (viral factory) in juxta-nuclear region manifested as a homogeneous, low-to-middle electron density, void of any organelles. The replication begins with formation of crescent (Cr), then immature virus (IV), immature virus with nucleoids (IVN), mature virus (MV). Many MV are wrapped by intracellular membrane from Trans-Golgi cistern, form intracellular enveloped virus (IEV). IEVs move towards cell periphery, fuss with cell membrane and obtained another envelope from cell membrane, finally they are released to extracellular space to form extracellular enveloped virus (EEV). Some EEV remain attached on cell membrane as cell associated extracellular enveloped virus (CEV). **C-D** Different intermediate particles from cells other than cells in a-b in high magnification.

Findings from electron microscopy sections of infected cells are in agreement with the findings from negative stained whole mount viral particles, and provided more details about the maturation status of the viral particles. Infected A549 cells produce the whole range of viral particles at different maturation stages. A low magnification outlook of two adjacent cells (**Fig. 4A**) showing numerous viral particles, most of them are mature virus (MV), majority of the MV are wrapped or enveloped intracellular enveloped virus (IEV) by membrane from trans-Golgi cistern, and the extracellular viral particles are wrapped by double membrane (EEV) with outer membrane from cell plasma membrane. Some EEV are still associated with cells (CEV). Inserts with high magnification show them with clear membrane layers. Fig. 4B shows the viral factory in the lower left cell in Fig. 4A. The viral factory (early replication center) is manifested by a low-to-middle electron density area in the juxta-nuclear region void of any cellular organelles. Intracellular enveloped viruses (IEV) come out farther from the factory. Fig. 4C and 4D show another viral factory and wrapped vaccinia viral particles inside or outside the cell in higher magnification.

**Fig 5:**
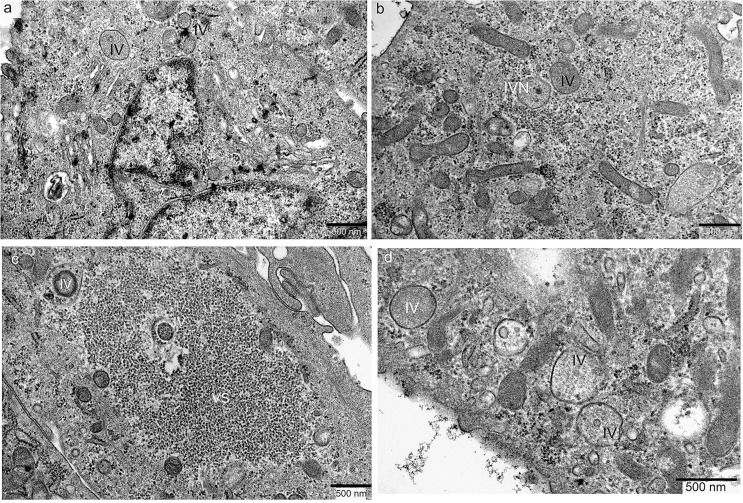
Abortive vaccinia virus replication and maturation in SCCF1 cells. **A)** At 48 hours after infection, the replication centre (viral factory) is inconspicuous, only a few immature viruses (IV) were found around it. **B)** Two IVs were visible, one IV showed nucleoid inside thus can be identified as IVN. Neither mature viruses nor enveloped viruses were detected throughout all infected cells. **C)** there are two small immature viruses (IV) located within cell and in the edge of an abnormal granular virosome (VS) region. No MVs were visible there. **D)** The immature viruses (IV) migrate to peripheral of the cell, underneath cell membrane. They still fail to get membranous envelope even at this late stage of nearly being released out.

Inside the infected SCCF1 cells, the picture was different: the replication factory is inconspicuous, there were just some immature viruses (IV) of different size (**Fig. 5A**), and a few of them have DNA injected to form nucleoids (IVN) (**Fig. 5B**). In some areas, the replication factory contains abnormal granular virosomes (VS), with a few IVs scattered around (**Fig. 5C**). Even at the peripheral region, where viruses are going to be release outside of the cells, there were only enlarged IVs inside cytoplasm and no membranous envelope obtained. Also, no EEVs were found outside the cells (**Fig. 5D**).

## Discussion

The life cycle of Vaccinia virus is well characterized starting from the moment it enters the cell [14]. Vaccinia virus replication takes place in cytoplasm, where virions are fully uncoated by viral enzymes released from the virion core. The viral DNA is not infectious per se. The replication cycle can be divided into functions controlled by early gene products and those controlled by late gene products [15].

Conventionally, it has been perceived that vaccinia virus produces four different types of virions from each infected cell. Most virions remain within cells thus lacking the outer envelope found on released virions. These intracellular mature viruses (IMV) stay inside cells until lysis; constituting robust, stable virions well suited for transmission of infection between hosts. Intracellular enveloped virus (IEV) is formed by wrapping of IMV with intracellular membranes, and is an intermediate between IMV and cell-associated enveloped viruses (CEV) enabling efficient virus dissemination to the cell surface, utilizing microtubules [16]. CEV induces the formation of actin tails that drive CEV particles away from the cell which is important for cell-to-cell spread [17]. Extra-cellular enveloped virus particles (EEV) which contain an additional host-cell derived envelope mediate the long-range dissemination of virus in the bloodstream [18]. The production of several different particle forms during the replication cycle secures efficient spread of the virus and helps virus to evade antibodies and complement [19–21].

Several virus-encoded proteins have been identified to have a role in virus morphogenesis. For example, the VV encoded I7 protein participates in proteolytic processing of the vaccinia virus membrane and core components, and is critical for transition from IVN to MV [22]. Lack of I7 results in arrest of VV morphogenesis with some similarity to what was observed in the SCCF1 cell line in our experiments. Other characterized virus proteins affecting morphogenesis include for example kinases B1 and F10 and a protein phosphatase H1. Moreover, phosphorylation of several key poxvirus proteins, including A14, A30 and G7, is required for early morphogenesis, and deletion of F10 results in lack of virosome condensation and crescent formation [23, 24].

As mentioned above, the viral protein defects affect replication, maturation and morphogenesis of vaccinia virus. However, our data suggests that the similar effects might also be brought out by the host cells. It seems that the vaccinia viral particles within SCCF1 cells were not able to form the core unit from viral DNA and thus no MV was detected. One possible explanation is that although the crescent can close to form IV, the viral genome DNA is not packed or condensed to form the core unit. The loose nucleosome threads need some core nucleoproteins to get further condensation and encapsidation. The IVN to MV transition appeared to be completely arrested in SCCF1 cells, as we did not see any MV by electron microscopy. However, it is important to keep in mind that thin section is not completely conclusive as a very small fraction of the cell volume is being visualized in each case. As all the core proteins and membrane proteins needed for virion maturation are encoded by the viral genome, it seems that some cellular co-factor target of a viral protein affecting the replication of the virus is missing or defective in the SCCF1 cell line. Alternatively, the cells might produce some anti-viral cytokines or interferons which can arrest the life cycle of the virus. Identifying the mechanism would yield interesting insight into both viral and cellular biology.

Notably, not much has been reported about vaccinia and feline cell lines. For domestic cats more than 400 cases of Cowpox virus (CPXV) infections have been described [25], but it is possible that many infections are not recognized by veterinarians or their owners. Cat-to-cat transmission is apparently rare [26]. Cats as predators are exposed to CPXV while hunting rodents, which serve as a reservoir for CPXV [27] and at least one such case of has been reported [28]. While cowpox replicates and causes disease in cats, vaccinia virus infection appears self-limiting and vaccinia virus (Lister strain) showed low infectivity in cats [26]. Poxviruses comprise a group of pathogens including some zoonotic members affecting livestock and humans. There might be genetically and phenotypically different poxvirus populations circulating in unknown natural reservoirs but the poxvirus maintenance in nature and their transmission to humans remains unknown [29]. Previously rabbit poxvirus, myxoma virus, has been shown to cause mild cytopathic effects in the SCCF1 line. Similar findings to ours were observed in this study; although cytopathic effects were seen, the replication of the virus was significantly slower than in positive control cell line and there was no statistically significant difference in cell death compared to mock-infected cells [30]. Further studies to determine if other feline neoplasms are susceptible to vaccinia virus infection are needed to demonstrate conclusively these findings.

To our knowledge, this is the first time this kind of replication pattern of vaccinia virus has been reported. These findings could provide valuable insights into molecular mechanisms underlying host cell infection, viral replication as well as virulence and life cycle of vaccinia virus.

## Supporting Information

S1 Fig Transduction of vvdd in SCCF1 cell line. Infectious virus was recovered from SCC-F1 cells by plaque forming test after infection of cells with 0.01 pfu/cell.
(TIF)

S2 Fig Normal ultrastructures of SCCF1 cells. **A)** The SCC cell line was derived from squamous cell carcinoma and maintains most of the morphological features of squamous epithelial cells; nucleus with loosely packed chromatin (Nu), cytoplasm is rich in different organelles like mitochondria (Mit), ribosomes and endoplasmic reticulum, patches of glycogen (gl), and most prominently, bundles of tonofibrils (Tf) consisting of keratin intermediate filaments. **B)** Cells also display characteristics of cancer cells such as large nucleus filled with low electron density euchromatin (Nu) and prominent nucleolus (No). **D)** Mitochondria within the cells were in a high dynamic process of fusion and fission resulting in numerous long, irregular, branching mitochondria, phenomena common when cells are under stress.
(TIF)

S1 Table Colony counts of clonal assay. SCCF1 cells were infected with vvdd-tdTomato 10 pfu/cell in duplicates and 10,000, 1,001, 100 or 10 infected cells were grown 1 or 3 days to evaluate if cell were able to form colonies.
(DOCX)
