# Supplementary figures and images for: Incomplete but Infectious Vaccinia Virions Are Produced in the Absence of Oncolysis in Feline SCCF1 Cells
Source: PLoS One. 2015 Mar 23;10(3):e0120496. doi: 10.1371/journal.pone.0120496 (PMC4370597; doi:10.1371/journal.pone.0120496)

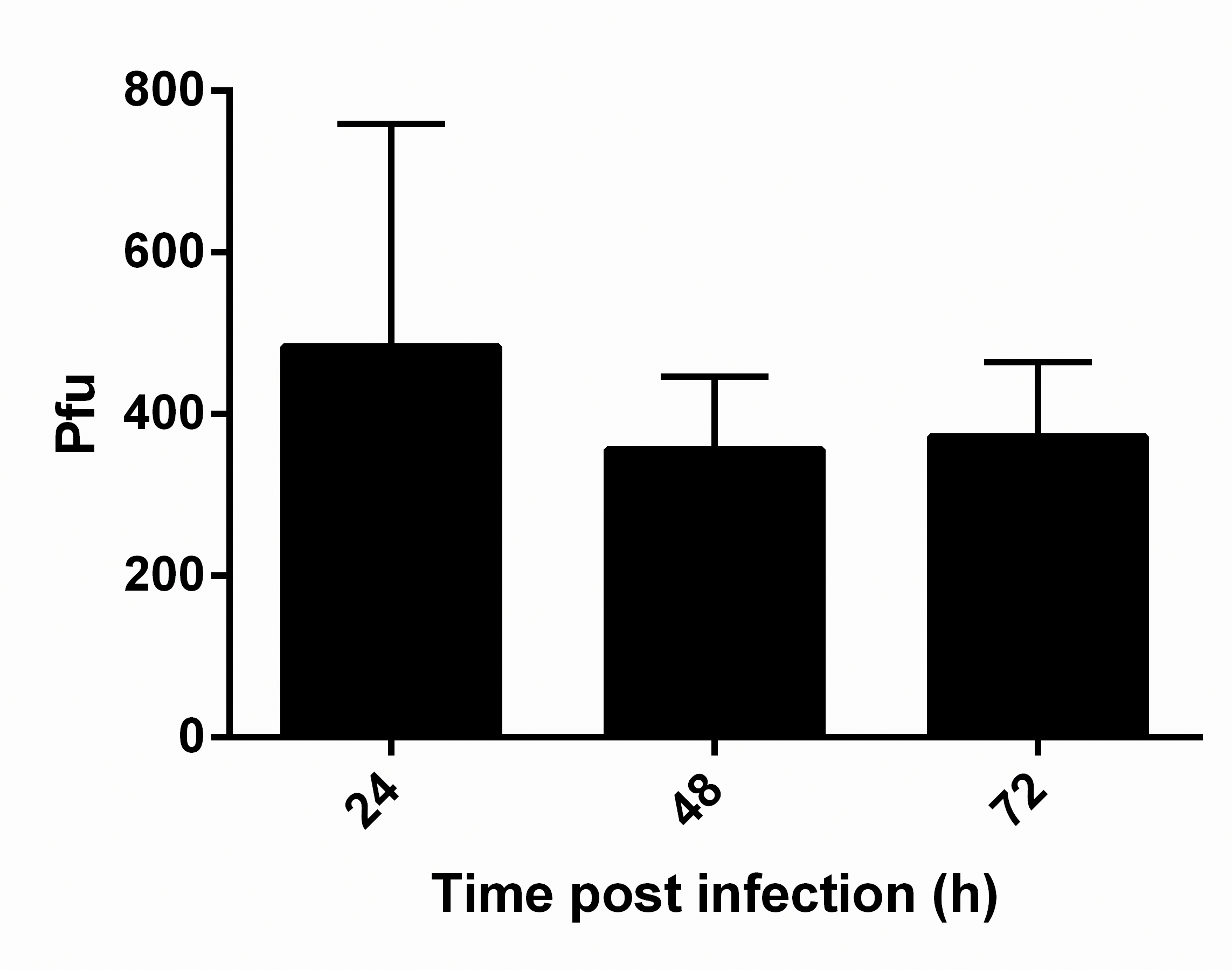

Supplement: S1 Fig — Infectious virus was recovered from SCC-F1 cells by plaque forming test after infection of cells with 0.01 pfu/cell. (TIF) [file pone.0120496.s001.tif]

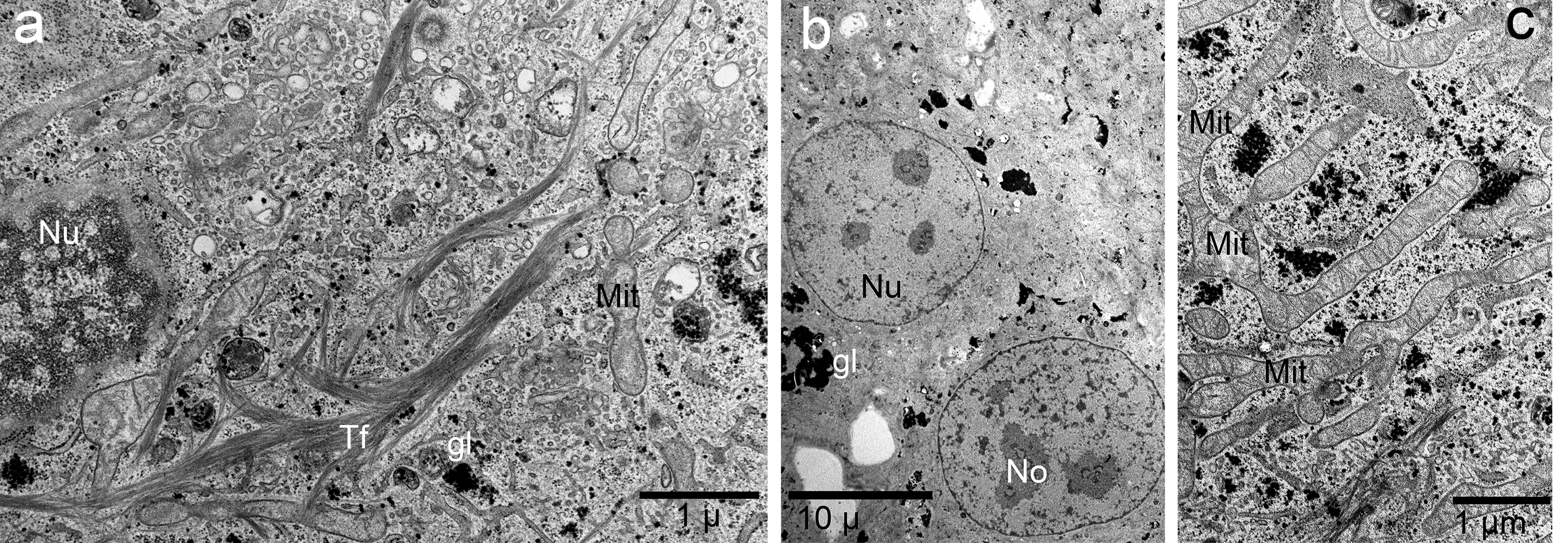

Supplement: S2 Fig — A) The SCC cell line was derived from squamous cell carcinoma and maintains most of the morphological features of squamous epithelial cells; nucleus with loosely packed chromatin (Nu), cytoplasm is rich in different organelles like mitochondria (Mit), ribosomes and endoplasmic reticulum, patches of glycogen (gl), and most prominently, bundles of tonofibrils (Tf) consisting of keratin intermediate filaments. B) Cells also display characteristics of cancer cells such as large nucleus filled with low electron density euchromatin (Nu) and prominent nucleolus (No). D) Mitochondria within the cells were in a high dynamic process of fusion and fission resulting in numerous long, irregular, branching mitochondria, phenomena common when cells are under stress. (TIF) [file pone.0120496.s002.tif]
