# Supplementary material for: Incomplete but Infectious Vaccinia Virions Are Produced in the Absence of Oncolysis in Feline SCCF1 Cells
Source: PLoS One. 2015 Mar 23;10(3):e0120496. doi: 10.1371/journal.pone.0120496 (PMC4370597; doi:10.1371/journal.pone.0120496)
Supplement: S1 Table — SCCF1 cells were infected with vvdd-tdTomato 10 pfu/cell in duplicates and 10,000, 1,001, 100 or 10 infected cells were grown 1 or 3 days to evaluate if cell were able to form colonies. (DOCX) [file pone.0120496.s003.docx]

# Tables

**Table S1.** **Colony counts of clonal assay.**

| **Time** | **Treatment** | **Number of cells plated** | | |  |  | |  | |
| --- | --- | --- | --- | --- | --- | --- | --- | --- | --- |
|  |  | 10,000 | 1,000 | 100 | | | | 10 | |
|  |  | **Number of cell colonies** | | |  | |  | |  |
| Day 1 | Mock | Max | 159 | 29 | | | | 4.0 | |
|  | vvdd-tdTomato | Max | 43.3 | 3.3 | | | | 0 | |
| Day 3 | Mock | Max | 187 | 10.6 | | | | 2.1 | |
|  | vvdd-tdTomato | Max | 102 | 7.5 | | | | 1.5 | |

Max indicates too many colonies to be counted
